# Cryptochrome 1 in Retinal Cone Photoreceptors Suggests a Novel Functional Role in Mammals

**DOI:** 10.1038/srep21848

**Published:** 2016-02-22

**Authors:** Christine Nießner, Susanne Denzau, Erich Pascal Malkemper, Julia Christina Gross, Hynek Burda, Michael Winklhofer, Leo Peichl

**Affiliations:** 1 Max Planck Institute for Brain Research, Max-von-Laue-Str. 4, 60438 Frankfurt am Main, Germany; 2 Department of Earth and Environmental Sciences, Ludwig-Maximilians-University Munich, Theresienstr. 41, 80333 Munich, Germany; 3 Ernst Strüngmann Insitute for Neuroscience, Deutschordenstr. 46, 60528 Frankfurt am Main, Germany; 4 Department of Biosciences, Goethe University Frankfurt am Main, Max-von-Laue-Str. 13, 60438 Frankfurt am Main, Germany; 5 Department of General Zoology, Faculty of Biology, University of Duisburg-Essen, Universitätsstr. 5, 45141 Essen, Germany; 6 Department of Game Management and Wildlife Biology, Faculty of Forestry and Wood Sciences, Czech University of Life Sciences, Kamýcká 129, 165 21 Praha 6 - Suchdol, Czech Republic; 7 Department of Hematology/Oncology and Developmental Biochemistry, University Medicine, Justus-von-Liebig-Weg 11, 37077 Göttingen, Germany; 8 Institute for Biology and Environmental Sciences IBU, University of Oldenburg, 26111 Oldenburg, Germany

## Abstract

Cryptochromes are a ubiquitous group of blue-light absorbing flavoproteins that in the mammalian retina have an important role in the circadian clock. In birds, cryptochrome 1a (Cry1a), localized in the UV/violet-sensitive S1 cone photoreceptors, is proposed to be the retinal receptor molecule of the light-dependent magnetic compass. The retinal localization of mammalian Cry1, homologue to avian Cry1a, is unknown, and it is open whether mammalian Cry1 is also involved in magnetic field sensing. To constrain the possible role of retinal Cry1, we immunohistochemically analysed 90 mammalian species across 48 families in 16 orders, using an antiserum against the Cry1 C-terminus that in birds labels only the photo-activated conformation. In the Carnivora families Canidae, Mustelidae and Ursidae, and in some Primates, Cry1 was consistently labeled in the outer segment of the shortwave-sensitive S1 cones. This finding would be compatible with a magnetoreceptive function of Cry1 in these taxa. In all other taxa, Cry1 was not detected by the antiserum that likely also in mammals labels the photo-activated conformation, although Western blots showed Cry1 in mouse retinal cell nuclei. We speculate that in the mouse and the other negative-tested mammals Cry1 is involved in circadian functions as a non-light-responsive protein.

Cryptochromes are blue-light absorbing flavoproteins that in animals have an important function in the circadian clock^1^. Furthermore, cryptochromes have been suggested to act as receptor molecules with magnetically sensitive radical pair reactions in the light-dependent magnetic compass sense of birds^2^. This is supported by experimental evidence^3^,^4^,^5^,^6^, although the precise mechanism of magnetic signaling remains to be elucidated.

For cryptochrome as a globular protein to act as an effective magnetic sensor for magnetic field directions according to the Radical Pair Model^2^,^7^, several structural requirements have to be met: (i) an anchor to provide a temporally stable orientation against Brownian motion, (ii) a cellular structure that orients the anchored proteins coherently within the receptor cell, (iii) an arrangement of receptor cells that covers all spatial directions. Cryptochrome 1a (Cry1a) is bound to the disk membranes of all UV/violet-sensitive S1 cones in the retina of European robins (*Erithacus rubecula*) and chickens (*Gallus gallus*)^8^. This localization makes Cry1a a good candidate for the receptor molecule of the avian magnetic compass, because the stacked disk membranes provide a cell-fixed axis, and the S1 cones are distributed across the entire retinal hemisphere. Furthermore, photo-activated Cry1a occurs under those spectral light conditions that also evoke behavioral magnetic responses^9^,^10^.

Birds have two splice products of the *Cry1* gene, Cry1a and Cry1b^11^. The putative magnetoreceptor Cry1a of birds is homologous to mammalian Cry1^1^,^11^. The localization of Cry1 in the mammalian retina has been little studied to date. In the retina of laboratory mice, *Cry1* expression was detected in bipolar, horizontal, amacrine and ganglion cells but not in photoreceptors^12^,^13^. In contrast, photoreceptors of Wistar rats were reported to express *Cry1*^14^. Most mammals have two spectral cone photoreceptor types, longwave-sensitive L cones and shortwave-sensitive S1 cones, enabling dichromatic color vision (reviews^15^,^16^,^17^). Here, we were interested in retinal Cry1 localization across mammals and whether there is a common phylogenetic or ecological expression pattern. For this, we used an antiserum against the chicken Cry1a C-terminus that in birds has been shown to label only the photo-activated conformation^9^.

## Results

**Figure 1:**
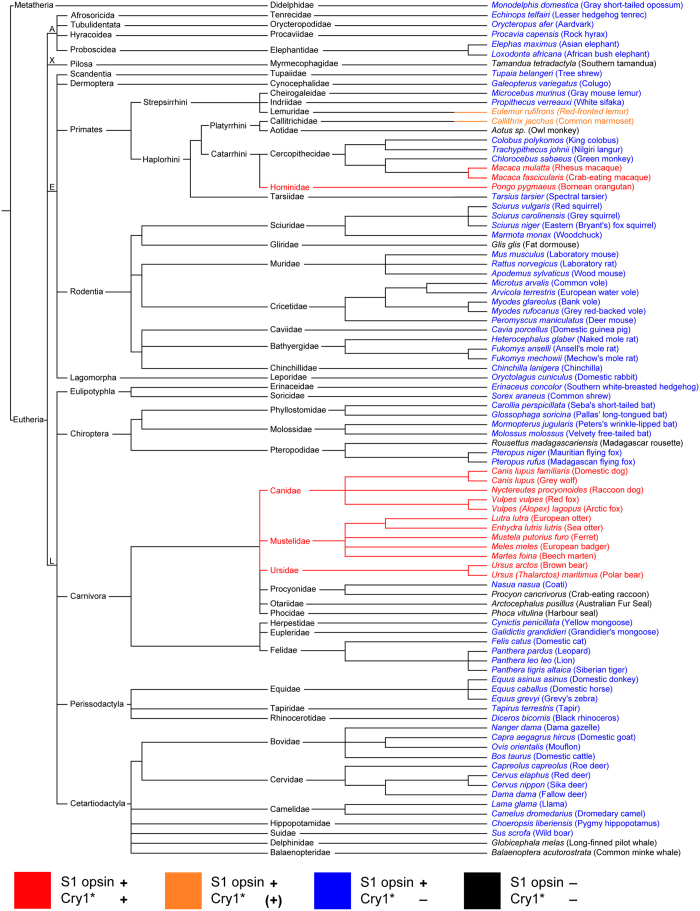
Expression pattern of Cry1* in the retinal S1 cones of the studied mammalian species. Color codes are given below the figure. The species are ordered according to their phylogenetic relationships^38^,^39^,^40^,^41^,^42^, branch lengths are not scaled to time. Cry1* label is only present in canoid carnivores and in some primates (species shown in red), and it is restricted to the outer segments of the S1 cones. In two primates Cry1* label is faint (shown in orange). Most species possess S1 cones but show no Cry1* label (blue). Some species do not possess S1 cones and show no Cry1* label (black). A more detailed tree of Carnivora is given in Supplementary Fig. S1. A, Afrotheria; X, Xenarthra; E, Euarchontoglires; L, Laurasiatheria.

**Figure 2:**
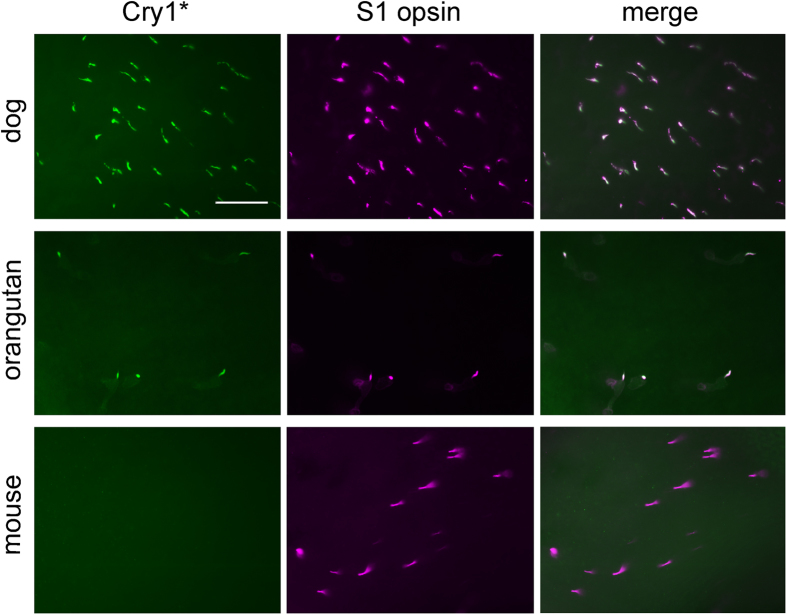
Cry1* label in the S1 cones of the dog and orangutan retina but not in the mouse retina. Images of the photoreceptor layer in retinal flatmounts of dog, orangutan and mouse. Left column: Cry1* immunofluorescence (green) is present in dog and orangutan retina, but not in mouse retina. Middle column: S1 cone opsin immunofluorescence (magenta) in the same areas (frames). Right column: Merged images, showing that Cry1* and S1 cone opsin co-localize in the dog and orangutan retina. The scale bar represents 50 μm and applies to all panels.

Our immunohistochemical double-labeling for the potentially light-activated conformation of Cry1 (here termed Cry1*) recognized by the used antiserum, and for the shortwave-sensitive S1 cone opsin in the retinae of 90 species across 48 families in 16 mammalian orders revealed a non-uniform pattern (Fig. 1). In the majority of taxa, no Cry1* label was detected; only in three Carnivora families and some Primates of different families, Cry1* was labeled, localized in the outer segment of the S1 cones. Three examples are shown in Fig. 2. In the dog (*Canis lupus familiaris*) retina, Cry1* was present in all cones containing S1 opsin, as best seen in retinal flatmounts (Fig. 2 top). The same held true for the hominid orangutan (*Pongo pygmaeus*) retina (Fig. 2 middle). In contrast, in the laboratory mouse (*Mus musculus*) retina no Cry1* was visible (Fig. 2 bottom). Supplementary Table S1 shows an example of every species we tested.

Among the Carnivora, we found Cry1* located in the S1 cone outer segments of all studied Canidae, Mustelidae and Ursidae, whereas the Felidae, Procyonidae, and the marine Phocidae and Otariidae were negative for Cry1* (Supplementary Figs. S1, S2; Supplementary Table S1). In primates, the results were less uniform. The orangutan, the rhesus macaque (*Macaca mulatta*) and the crab-eating macaque (*Macaca fascicularis*) showed S1 cone-selective strong Cry1* labeling comparable to that in the Canidae (Fig. 2, Supplementary Fig. S3). In the red-fronted lemur (*Eulemur rufifrons*; Supplementary Fig. S3) and the common marmoset (*Callithrix jacchus*), the Cry1* labeling intensity was markedly lower but still restricted to the S1 cones. The crab-eating macaque (*Macaca fascicularis*) showed, in addition to the label in S1 cones, Cry1* label in some outer segment-shaped structures that did not colocalize S1 opsin or longwave-sensitive L opsin label (Supplementary Fig. S3). These sparse and unevenly distributed Cry1*-positive structures could be degenerated or damaged photoreceptors. In the green monkey (*Chlorocebus sabaeus*) (Supplementary Fig. S3), as in the other strepsirrhine and haplorrhine monkeys studied, there was no Cry1* label (Supplementary Table S1).

Negative for Cry1* were, amongst others, all studied Rodentia, Perissodactyla, Artiodactyla, Chiroptera and Proboscidea (Supplementary Fig. S2, Supplementary Table S1). S1 cones are absent in all Cetacea, Phocidae and Otariidae, and in some species from other groups^17^,^18^; no Cry1* could be detected in the retinae of these species. In none of the 90 species studied, Cry1* label was seen in any other retinal cell type than S1 cones (Supplementary Fig. S2), except for *Macaca fascicularis* (see above). Incidentally, for some of the species listed in Fig. 1 and Supplementary Table S1, this is the first description, to our knowledge, of the presence of S1 cones in their retinae.

Given the high inter-species sequence homology of the Cry1 protein epitope recognized by our antiserum (Supplementary Table S2), we wondered about the absence of Cry1* immunolabeling in the majority of species. This was addressed exemplarily with Western blotting of cell fractionated retinae of dog and mouse (Supplementary Fig. S4). In the dog, Cry1 was found in the cytosolic and membrane fraction. In the mouse, Cry1 was also detectable, but in other cell compartments, namely the membrane and nucleus fraction. Since the Western blotting was performed on fractionated whole retinae, these results do not indicate in which retinal cell types the detected Cry1 was located.

## Discussion

We tested a broad range of mammals for the localization of Cry1 in the retina, using an antiserum that in birds only recognizes the light-activated conformation of Cry1a. For clarity we use the term Cry1* for the mammalian Cry1 conformation labeled by the antiserum. To our knowledge, this is the first histological assessment of the retinal localization of the Cry1 protein in any mammal. For some of the species, it also is the first report of S1 cones in their retinae. We found Cry1* label only in Canidae, Mustelidae, Ursidae, and in some Primates; in these species the Cry1* label was exclusively seen in the S1 cones, not in any other cell type (except for *Macaca fascicularis*). The unexpected differences of Cry1* labeling across mammals suggest corresponding differences in Cry1 functions. The systematic analysis reveals no correlation of the Cry1* labeling with the species’ diel activity patterns (nocturnal, diurnal, crepuscular, arrhythmic) or habitats; the only obvious correlation is with phylogenetic grouping.

The amino acid sequence used as antigen for the production of our antiserum is part of the chicken Cry1a C-terminal region. It also matches the Cry1 sequence of the mammalian species showing no immunolabel in intact tissue (Supplementary Table S2). In the Western blots, however, the antiserum detected Cry1 also in the mouse, where it was located in the nucleus fraction. In chicken we recently showed that our antiserum binds the avian Cry1a only in its light-activated conformation^9^, where the C-terminal domain becomes exposed at the protein surface^19^,^20^,^21^,^22^. Thus, mouse Cry1 probably is not detected in the tissue because it is not present in the light-activated conformation. This also would explain why, in contrast to an earlier report^14^, we did not detect Cry1 in the photoreceptors of the rat. During cell fractionation for Western blotting, the protein loses its conformation because of the denaturing conditions, providing access to the C-terminus. The retina is part of the mammalian circadian clock, where Cry1 acts as a light-independent negative limb in the nuclear circadian feedback loop^1^. We assume that in the species with no immunohistochemical Cry1* labeling, Cry1 is not light-responsive and has transcription-regulating functions in the circadian system.

In the absence of definitive evidence, it is a parsimonious assumption that also in mammals, the conformation Cry1* is activated by light. For most, but not all of the species studied here we know that the animals were exposed to light before they died, and that the eyes were excised and fixed at light. In fact, after dark adaptation bird Cry1a only needs 5 minutes of light exposure to re-acquire the light-activated conformation recognized by the antiserum^9^. To conclusively show that mammalian Cry1* also is a light-activated conformation, one would have to demonstrate the absence of labeling with our antiserum in dark adapted Canoidea and Primates.

What function can be surmised for Cry1* in those species where it is found in the outer segment of the S1 cones? This location is not in accord with a role in nuclear transcription regulation, for which a perinuclear location would be expected. Hence we dismiss a function in the circadian clock. The location in the outer segment would be compatible with a visual function, similar to that of visual pigments. Cryptochrome can undergo a light-induced conformation change, although it is unknown whether it is linked to a transduction cascade (e.g., a transducin-like G protein). If Cry1* acts as a visual pigment, it is expected to widen the spectral sensitivity of the S1 cones. Cryptochromes are sensitive to light from the UV to the green part of the spectrum^1^,^23^, whereas mammalian S1 pigments are more narrowly tuned to blue, violet or UV, depending on species (review^17^). In many mammals UV light is absorbed by the lens and does not reach the retina, but a number of mammalian species have UV-transparent lenses even though they have violet- or blue-sensitive S1 cones^24^. In the latter species, the S1 cones can detect UV by the shortwave flank of the S1 pigment tuning curve. However, our sample shows no correlation between Cry1* label, tuning of the S pigment and UV transparency of the lens. Both dog and cat have a blue-tuned S1 pigment and a UV-transparent lens, but the dog shows Cry1* label whereas the cat does not. The diurnal squirrels have UV-absorbing lenses and the nocturnal murids UV-transparent ones, but both groups of rodents show no Cry1* in their S1 cones.

Another possible function is magnetoreception similar to that proposed for the avian magnetic compass. In animals in general, two basic mechanisms are discussed for the detection of the geomagnetic field: one based on spin-correlated radical pairs probably generated by Cryptochrome (reviews^6^,^25^), the other based on ferrimagnetic particles like magnetite^26^. It is intriguing that we found Cry1* label in S1 cones only in two phylogenetically distinct groups, the Canoidea (caniform carnivores) and Primates. In both groups, behavioral studies indicate responses to the magnetic field. Among the Canidae, magnetic alignment has been suggested for red fox and dog (reviews^27^,^28^); among Primates, humans are discussed to be able to detect the Earth’s magnetic field^29^ (review^28^). However, it is unknown what kind of mechanism underlies their magnetic responses. Furthermore, magnetic alignment has also been observed in mammals that do not show Cry1* label in their S1 cones, e.g., artiodactyls^27^,^28^.

Among rodents, responses to the magnetic field have been demonstrated in several epigeic^30^,^31^ and subterranean species^32^. The detection mechanism in epigeic rodents has been discussed to be a radical pair mechanism^30^,^31^,^33^. However, the absence of retinal Cry1* label in all epigeic rodents studied here, including the taxa and species with behaviorally demonstrated magnetic responses, suggests a reception mechanism different from that of birds. The magnetic sense of subterranean mole-rats^32^ and of bats^34^ is not light sensitive and probably based on magnetite rather than a radical pair mechanism^28^. In line with this, we did not find Cry1* label in mole-rat and bat retinae.

At present, the data base is not sufficient to suggest a correlation between the occurrence of Cry1* in the cones and magnetoreception mechanisms in mammals. Future studies will have to elucidate whether there is a link between light-activated Cry1 in mammalian S1 cone outer segments and magnetoreception and/or vision. A further question is how an animal could separate visual and magnetic information when they are signaled via the same photoreceptors. This is open even for the bird S1 cones (for a discussion, see^35^).

## Methods

### Tissue preparation

The retinae of 90 mammalian species were used for this study. Some animals had been sacrificed in the laboratory for unrelated experiments, some eyes came from animals that had died in captivity, some from hunted animals, and some from road kills. For most species, fixed eyes were sent to us by the colleagues who obtained them; for details see Supplementary Table S1. For animals from breeding colonies, all procedures for animal handling, experimentation and killing were in accordance with the relevant guidelines and regulations in the respective countries; compliance with the relevant guidelines and regulations was also observed for the animals obtained in the wild. For most non-laboratory species, only one or both eyes of one individual were available to us. To the best of our knowledge, all eyes were taken and fixed at daylight or under conventional laboratory lighting conditions. Enucleated eyes were opened by a cut around the cornea and commonly immersion-fixed in 4% paraformaldehyde in 0.1M phosphate buffer (PB, pH 7.4). In some instances the fixative was 4% formaldehyde in physiological saline. After washing out the fixative with several changes of PB, the retinae were isolated. Whole retinae or retinal pieces were cryoprotected in an ascending series of 10%, 20% and 30% sucrose in PB and frozen at −20 °C for storage. We used both cryosections and pieces of unsectioned retinae (flatmounts) for staining. Cryosections were oriented perpendicular to the retinal layers, they are termed ‘vertical sections’ here.

The onset of fixation after death (from 15 minutes to several hours), the duration of fixation (from 15 minutes to several days), the duration of storage at −20 °C (from a few days up to about two decades), and the state of the tissue differed between the animals. There were no noticeable differences in immunostaining associated with the different fixation parameters. For example, within the canid species that labeled for Cry1*, the full range of parameters was represented. Conversely, various fixation times starting immediately post mortem did not make any difference in the non-labeling of the mouse retina.

For Western blotting, eyes of light-adapted laboratory mice and a dog were obtained directly post mortem, the fresh unfixed retinae were isolated and immediately frozen at −80 °C for storage.

### Primary antibodies used for immunohistochemistry

The following antibodies were used:

1. Guinea pig Cry1a antiserum (designed in our laboratory and produced by GENOVAC GmbH, Freiburg, Germany), raised against amino acids 601–621 of Cryptochrome 1a in chicken: (N-) RPNPE EETQS VGPKV QRQST (-C), characterized in^8^. The amino acid sequence is very similar to that of Cry1 from different mammalian species (see Supplementary Table S2).
2. Goat antiserum sc-14363 raised against a 20-aa N-terminal epitope of the human S1 (blue) cone opsin (Santa Cruz Biotechnology Inc., Santa Cruz, CA, USA), characterized by^36^.
3. Rabbit antiserum JH 492 raised against the last 38 amino acids of the human L (red) cone opsin; kindly provided by J. Nathans, characterized by^37^.

### Immunohistochemistry

The retinae were pre-incubated with 10% normal donkey serum (NDS) in 0.25% Triton X-100, 2% bovine serum albumin (BSA) in PB for 60 min at RT and then incubated with a mixture of the primary antibodies (anti-Cry1 diluted 1:100, and sc-14363 diluted 1:500 or JH 492 diluted 1:2000) in 3% NDS, 0.25% Triton X-100, 2% BSA, in PB overnight. After washing in PB, the tissue was incubated with the donkey-anti-guinea pig, donkey-anti-goat and donkey-anti-rabbit secondary antibodies coupled to different fluorescent dyes (Alexa 488, Cy5, Cy3, DyLight 647; dilution 1:500; Dianova, Hamburg) in 3% NDS, 0.25% Triton X-100, 2% BSA, in PB for 1h. After staining, the retinae were coverslipped with Aqua-Poly Mount (Polysciences Europe) and evaluated at a confocal laser-scanning microscope (Zeiss LSM 510 META) or a Zeiss Axioplan 2 microscope.

The absence of Cry1* labeling in optimally fixed rodent retinae shows that there is no cross-reactivity of the Cry1 antiserum with the S1 cone opsin. The Cry1* labeling in suboptimally fixed Canoidea retinae shows that the labeling does not depend on narrowly specified tissue conditions.

### Western blot and cell fractionation

The cell fractionation was performed with the ProteoExtract® Subcellular Proteome Extraction Kit (Calbiochem) according to the manufacturer’s manual and as described previously^8^. In this differential cell fractionation protocol, the increasing dissolving strength of the buffers separates cytosolic, membrane, nuclear and cytoskeleton fractions of the retina. Then equal volumes of samples were subjected to 4–12% gradient SDS-polyacrylamide gel electrophoresis and electro-blotted onto nitrocellulose membrane for 1.5 hours at 180 mA. After blocking with 5% BSA for one hour, the membranes were incubated with the Cry1 antiserum (dilution 1:500) over night. Prestained plus protein marker (Thermo Scientific) was used for the size estimate of bands. The used fraction markers were: Protein Kinase C (dilution 1:1000; Santa Cruz Biotechnology Inc., Santa Cruz, CA, USA) for the cytosolic fraction, E-cadherin (dilution 1:1000; clone 36, BD Transduction Laboratories, Los Angeles, CA, USA) for the membrane fraction, Histone H3 (dilution 1:1000; Sigma, St. Louis, MO, USA) for the nuclear fraction, Actin (dilution 1:10,000; Sigma Aldrich, Munich, Germany) for the cytoskeletal fraction. Secondary antibodies, incubated for one hour, were horseradish peroxidase-conjugated goat anti-guinea pig, anti-rabbit and anti-mouse IgG antiserum, respectively (dilution 1:10,000; Dianova, Hamburg, Germany). Immunoblots were visualized using Immobilon Western Chemiluminescent HRP substrate (Merck Millipore) and are shown in Supplementary Fig. S4.

## Additional Information

**How to cite this article**: Nießner, C. *et al.* Cryptochrome 1 in Retinal Cone Photoreceptors Suggests a Novel Functional Role in Mammals. *Sci. Rep.* **6**, 21848; doi: 10.1038/srep21848 (2016).

## Supplementary Material

Supplementary Information
