# Author Correction: Cryptochrome 1 in Retinal Cone Photoreceptors Suggests a Novel Functional Role in Mammals

**DOI:** 10.1038/s41598-020-62295-2

**Published:** 2020-03-23

**Authors:** Christine Nießner, Susanne Denzau, Erich Pascal Malkemper, Julia Christina Gross, Hynek Burda, Michael Winklhofer, Leo Peichl

**Affiliations:** 1 Max Planck Institute for Brain Research, Max-von-Laue-Str. 4, 60438 Frankfurt am Main, Germany; 2 Department of Earth and Environmental Sciences, Ludwig-Maximilians-University Munich, Theresienstr. 41, 80333 Munich, Germany; 3 Ernst Strüngmann Insitute for Neuroscience, Deutschordenstr. 46, 60528 Frankfurt am Main, Germany; 4 Department of Biosciences, Goethe University Frankfurt am Main, Max-von-Laue-Str. 13, 60438 Frankfurt am Main, Germany; 5 Department of General Zoology, Faculty of Biology, University of Duisburg-Essen, Universitätsstr. 5, 45141 Essen, Germany; 6 Department of Game Management and Wildlife Biology, Faculty of Forestry and Wood Sciences, Czech University of Life Sciences, Kamýcká 129, 165 21 Praha 6, Suchdol, Czech Republic; 7 Department of Hematology/Oncology and Developmental Biochemistry, University Medicine, Justus-von-Liebig-Weg 11, 37077 Göttingen, Germany; 8 Institute for Biology and Environmental Sciences IBU, University of Oldenburg, 26111 Oldenburg, Germany

Correction to: *Scientific Reports* 10.1038/srep21848, published online 22 February 2016

This Article contains an error. Among the studied species, the orangutan was erroneously specified as Bornean orangutan *Pongo pygmaeus*. In fact, the studied individual was a Sumatran orangutan *Pongo abelii*.

In the Results section,

“The same held true for the hominid orangutan (*Pongo pygmaeus*) retina (Fig. 2 middle).”

should read:

“The same held true for the hominid orangutan (*Pongo abelii*) retina (Fig. 2 middle).”

The error is also present in Figure 1, the correct Figure 1 is given below.

**Figure 1:**
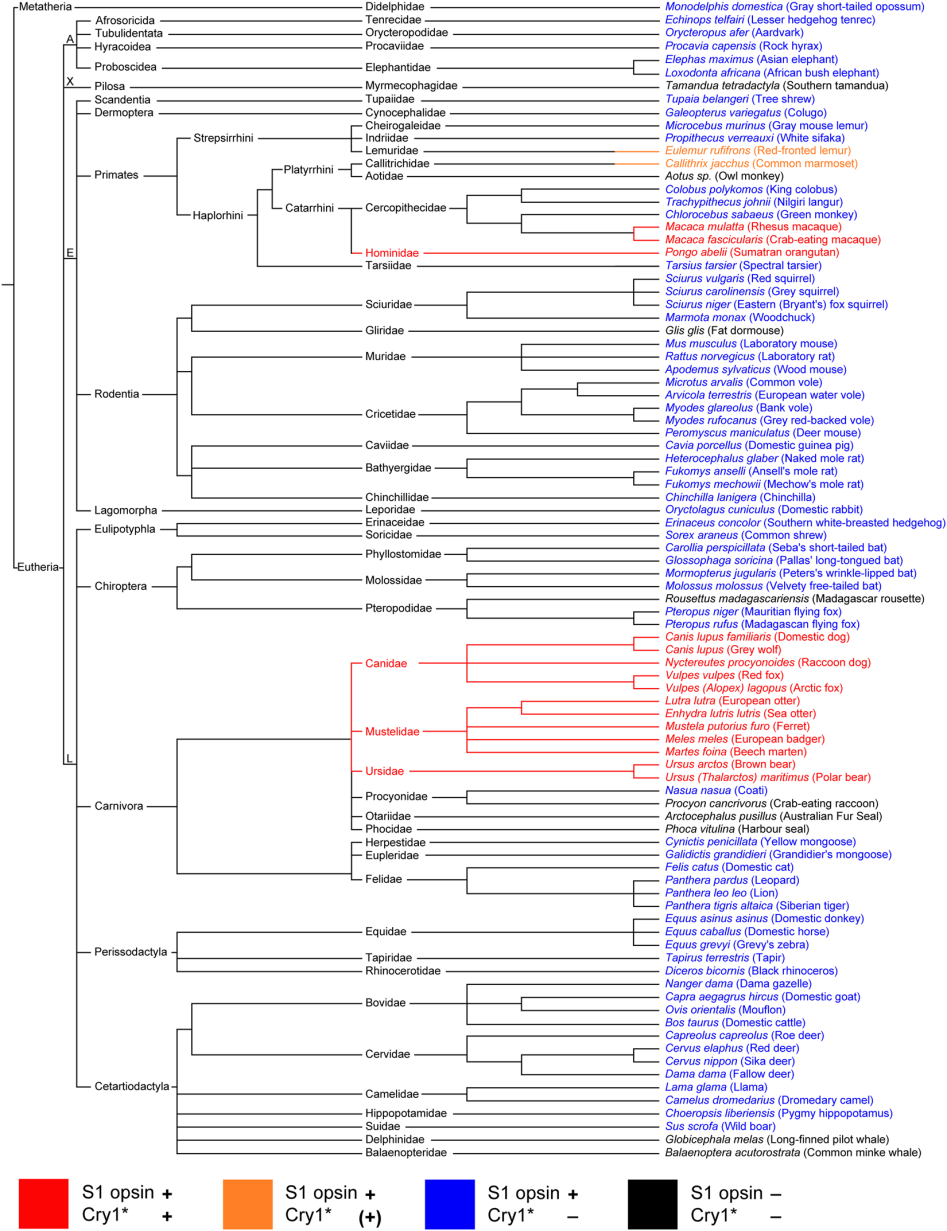
.

Finally, the error is present in Table S1, where

“Bornean orangutan, *Pongo pygmaeus*”

should read:

“Sumatran orangutan*, Pongo abelii*”

This error does not alter the conclusions of the paper.

