# Supplementary material for: Cryptochrome 1 in Retinal Cone Photoreceptors Suggests a Novel Functional Role in Mammals
Source: Sci Rep. 2016 Feb 22;6:21848. doi: 10.1038/srep21848 (PMC4761878; doi:10.1038/srep21848)
Supplement: Supplementary Information [file srep21848-s1.pdf]

Supplementary Information

Cryptochrome 1 in Retinal Cone Photoreceptors Suggests a Novel Functional Role in Mammals

Christine Nießner, Susanne Denzau, Erich Pascal Malkemper, Julia Christina Gross, Hynek Burda, Michael Winklhofer, Leo Peichl

Table S1. Retinal cryptochrome 1 and S1 opsin labeling in the species studied. The two left columns show representative high-resolution micrographs of the layer of photoreceptor outer segments in vertical retinal sections that had been double immunofluorescence-labeled for Cry1* (green) and S1 opsin (magenta). Each image pair shows exactly the same field, photographed with the respective fluorescence filters. Where Cry1* is labeled, it is located in the opsin-containing outer segments of the S1 cones. The right column gives the species names and tissue sources. Species are grouped taxonomically. Reference numbers refer to Supplementary References.

Cry1*	S1 opsin	Species
		**Metatheria**
		**Didelphimorphia**
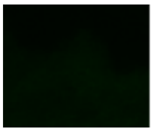	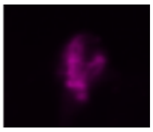	**Gray short-tailed opossum**, *Monodelphis domestica*<br>Adult, post mortem experimental material from breeding colony.<br>Courtesy L. Krubitzer & J. Luu, University of California Davis, CA, USA
		**Eutheria**
		**Afrosoricida**
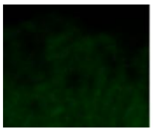	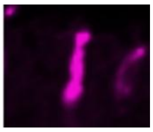	**Lesser hedgehog tenrec**, *Echinops telfairi*<br>Adult, post mortem experimental material from breeding colony.<br>Courtesy H. Künzle, Ludwig Maximilians University, Munich, Germany
		**Tubulidentata**
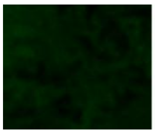	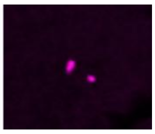	**Aardvark**, *Orycteropus afer*<br>Collection material. Courtesy P. Němec, Dept. of Zoology, Charles University, Prague, CZ
		**Hyracoidea**
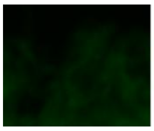	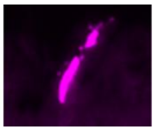	**Rock hyrax**, *Procavia capensis*<br>Collection material. Courtesy K. Moutairou, National University of Benin, Cotonou, Benin

Proboscidea

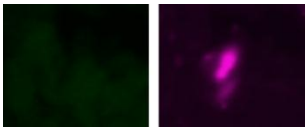

Asian elephant, *Elephas maximus*
Adult, autopsy material. Courtesy G. Wibbelt, Leibniz Institute for Zoo and Wildlife Research, Berlin, Germany

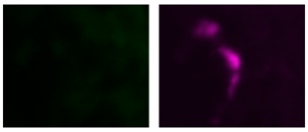

African bush elephant, *Loxodonta africana*
Adult, autopsy material. Courtesy G. Wibbelt, Leibniz Institute for Zoo and Wildlife Research, Berlin, Germany

Pilosa

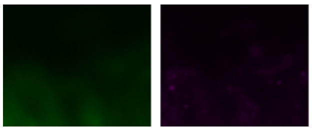

Southern tamandua, *Tamandua tetradactyla* (has no S1 cones; own observation)
Autopsy material. Courtesy G. Wibbelt, Leibniz Institute for Zoo and Wildlife Research, Berlin, Germany

Scandentia

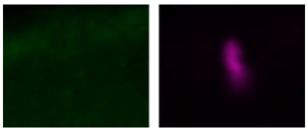

Tree shrew, *Tupaia belangeri*
Adult, post mortem experimental material from a breeding colony at MPI for Brain Research

Dermoptera

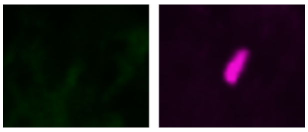

Colugo (flying lemur), *Galeopterus variegatus*
Wild adult found dead. Courtesy N. Lim, National University of Singapore & Raffles Museum of Biodiversity Research, Singapore

Primates

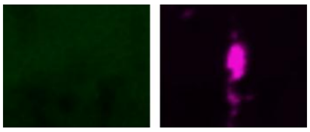

Gray mouse lemur, *Microcebus murinus*
Adult, post mortem experimental material. Courtesy A. Kaiser, Veterinary University Hannover, Germany

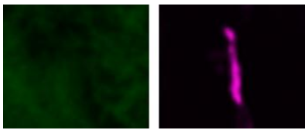

White sifaka, *Propithecus verreauxi*
Autopsy material. Courtesy P. Kappeler, German Primate Center Göttingen, Germany

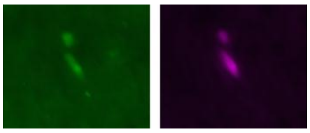

Red-fronted lemur, *Eulemur rufifrons*
Autopsy material. Courtesy P. Kappeler, German Primate Center Göttingen, Germany

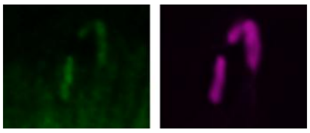

Common marmoset, *Callithrix jacchus*
Adult, post mortem experimental material. Courtesy C. Puller, MPI for Brain Research

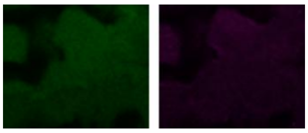

Owl monkey, *Aotus sp*. (has no S1 cones[1])
Adult female, post mortem experimental material. Courtesy A. Hendrickson, University of Washington, Seattle, WA, USA

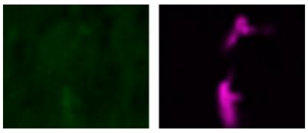

King colobus, *Colobus polykomos*
Adult, post mortem experimental material. Courtesy K. Mätz-Rensing, German Primate Center Göttingen, Germany

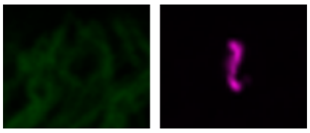

Nilgiri langur, *Trachypithecus johnii*
Adult, post mortem experimental material. Courtesy K. Mätz-Rensing, German Primate Center Göttingen, Germany

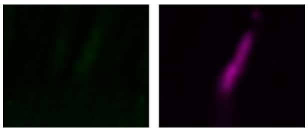

Green monkey, *Chlorocebus sabaeus*
Adult, post mortem experimental material. Courtesy R. Plesker, Paul-Ehrlich-Institut Langen, Germany

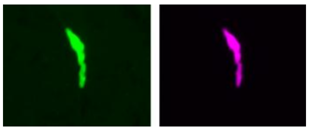

Rhesus macaque, *Macaca mulatta*
Adult, post mortem experimental material. Courtesy German Primate Center Göttingen, Germany

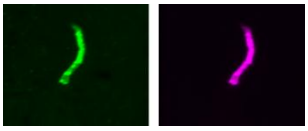

Crab-eating macaque, *Macaca fascicularis*
Old female, post mortem experimental material. Courtesy M. Munk, MPI for Brain Research

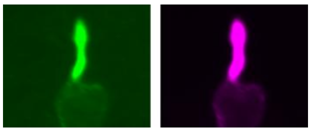

Bornean orangutan, *Pongo pygmaeus*
Old male, autopsy material. Courtesy Zoo Frankfurt/M., Morphisto GmbH and Senckenberg Museum Frankfurt/M., Germany

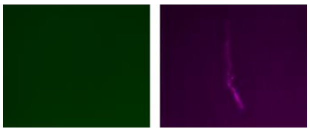

Spectral tarsier, *Tarsius tarsier*
Adult, post mortem experimental material. Courtesy A. Hendrickson, University of Washington, Seattle, WA, USA

Rodentia

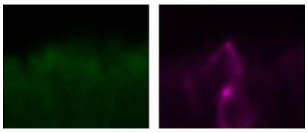

Red squirrel, *Sciurus vulgaris*
Eye collection of the MPI for Brain Research

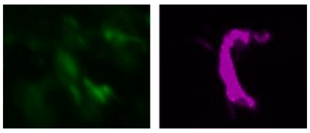

Grey squirrel, *Sciurus carolinensis*
Adult, culled animal. Courtesy UK Forestry Commission & G. Jeffery, University College London, UK

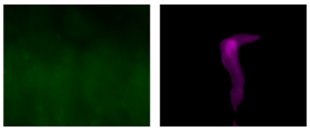

Eastern (Bryant's) fox squirrel, *Sciurus niger*
Post mortem experimental material. Courtesy N. Tararova, Medical School, Cleveland University, OH, USA

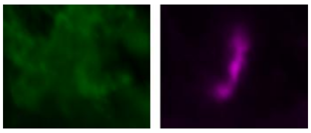

Woodchuck, *Marmota monax*
Post mortem experimental material. Courtesy P. Dammann, Central Animal Laboratory, Essen University Medical School, Germany

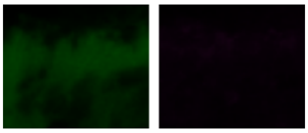

Fat dormouse, *Glis glis* (has no S1 cones[2])
Eye collection of the MPI for Brain Research

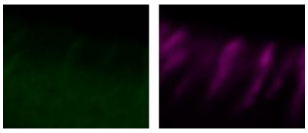

Laboratory mouse, *Mus musculus*
Adult, strain C57BL/6, breeding colony at MPI for Brain Research

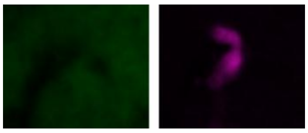

Laboratory rat, *Rattus norvegicus*
Adult, breeding colony at MPI for Brain Research

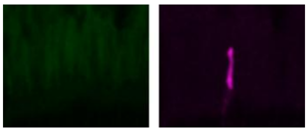

Wood mouse, *Apodemus sylvaticus*
Adult, killed for unrelated study at Dept. of General Zoology, University Duisburg-Essen, Germany

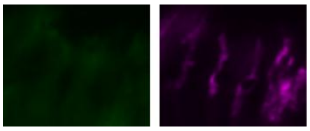

Common vole, *Microtus arvalis*
Courtesy S.A. Romanenko & A. Graphodaskii, Institute of Molecular and Cellular Biology, SB RAS, Novosibirsk, Russia

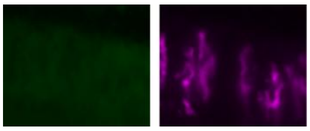

European water vole, *Arvicola terrestris*
Courtesy S.A. Romanenko & A. Graphodaskii, Institute of Molecular and Cellular Biology, SB RAS, Novosibirsk, Russia

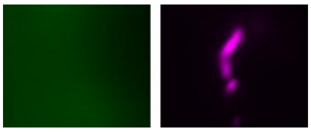

Bank vole, *Myodes (Clethrionomys) glareolus*
Courtesy S.A. Romanenko & A. Graphodaskii, Institute of Molecular and Cellular Biology, SB RAS, Novosibirsk, Russia

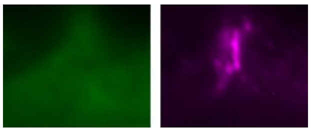

Grey red-backed vole, *Myodes* (*Clethrionomys) rufocanus*
Courtesy S.A. Romanenko & A. Graphodaskii, Institute of Molecular and Cellular Biology, SB RAS, Novosibirsk, Russia

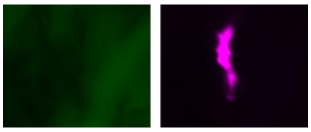

Deer mouse, *Peromyscus maniculatus*
Adult, breeding colony at MPI for Brain Research

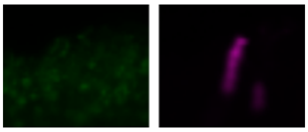

Domestic guinea pig, *Cavia porcellus*
Adult, from animal house at MPI for Brain Research

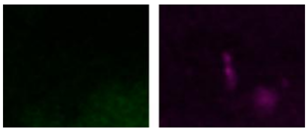

Naked mole rat, *Heterocephalus glaber* (subterranean)
Adult, killed for unrelated study at Dept. of General Zoology, University Duisburg-Essen, Germany

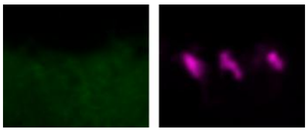

Ansell's mole rat, *Fukomys anselli* (subterranean)
Adult, killed for unrelated study at Dept. of General Zoology, University Duisburg-Essen, Germany

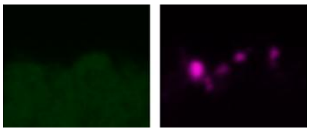

Mechow's mole rat, *Fukomys mechowii* (subterranean)
Adult, killed for unrelated study at Dept. of General Zoology, University Duisburg-Essen, Germany

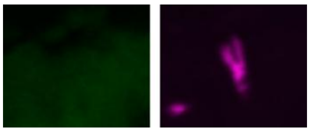

Chinchilla, *Chinchilla lanigera*
Adult, eye collection of the MPI for Brain Research

Lagomorpha

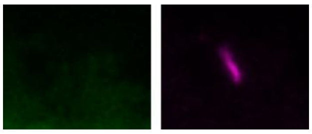

Domestic rabbit, *Oryctolagus cuniculus*
Adult, from animal house at MPI for Brain Research

Eulipotyphla

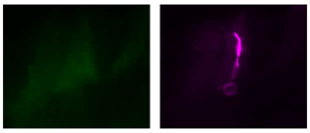

Southern white-breasted hedgehog, *Erinaceus concolor*
Courtesy S.A. Romanenko & A. Graphodaskii, Institute of Molecular and Cellular Biology, SB RAS, Novosibirsk, Russia

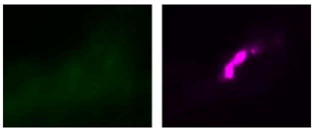

Common shrew, *Sorex araneus*
Courtesy S.A. Romanenko & A. Graphodaskii, Institute of Molecular and Cellular Biology, SB RAS, Novosibirsk, Russia

Chiroptera

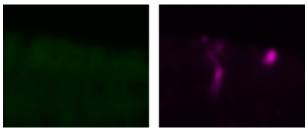

Seba's short-tailed bat, *Carollia perspicillata*
Post mortem experimental material. Courtesy B. Müller, MPI for Brain Research & M. Kössl, University Frankfurt/M., Germany

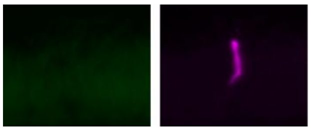

Pallas' long-tongued bat, *Glossophaga soricina*
Post mortem experimental material. Courtesy B. Müller, MPI for Brain Research

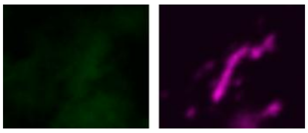

Peters's wrinkle-lipped bat, *Mormopterus jugularis*
Collection material. Courtesy S.M. Goodman, Field Museum of Natural History, Chicago, IL, USA, & WWF, Antananarivo, Madagascar

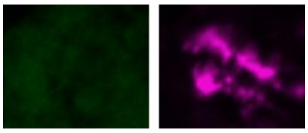

Velvety free-tailed bat, *Mollossus mollossus*
Post mortem experimental material. Courtesy B. Müller, MPI for Brain Research & M. Kössl, University Frankfurt/M., Germany

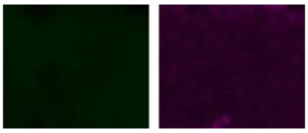

Madagascar rousette, *Rousettus madagascariensis* (has no S1 cones[3])
Collection material. Courtesy S.M. Goodman, Field Museum of Natural History, Chicago, IL, USA, & WWF, Madagascar

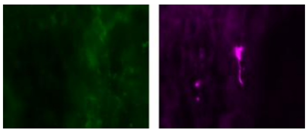

Mauritian flying fox, *Pteropus niger*
Collection material. Courtesy S.M. Goodman, Field Museum of Natural History, Chicago, IL, USA, & WWF, Madagascar

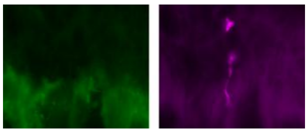

Madagascan flying fox, *Pteropus rufus*
Collection material. Courtesy S.M. Goodman, Field Museum of Natural History, Chicago, IL, USA, & WWF, Madagascar

Carnivora

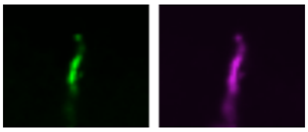

Domestic dog, *Canis lupus familiaris*
Adult Beagle-Setter-Mix, post mortem experimental material. Courtesy K. Stieger, Giessen University Eye Clinic, Giessen, Germany

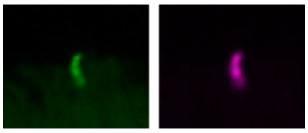

Grey wolf, *Canis lupus*
Adult, euthanized animal. Courtesy K. Burow, Wildlife Park 'Alte Fasanerie', Hanau, Germany

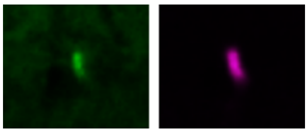

Raccoon dog, *Nyctereutes procyonoides*
Adult. Eye collection of the MPI for Brain Research

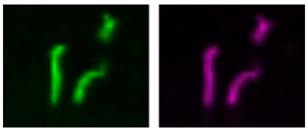

Red fox, *Vulpes vulpes*
Adult, hunted animal. Courtesy E. Noll, MPI for Brain Research

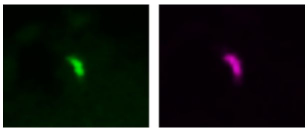

Arctic fox, *Vulpes (Alopex) lagopus*
Young adult. Eye collection of the MPI for Brain Research

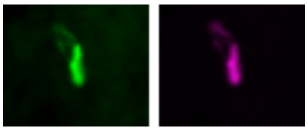

European otter, *Lutra lutra*
Adult, roadkill. Courtesy H. Ansorge, Staatliches Museum für Naturkunde, Görlitz, Germany

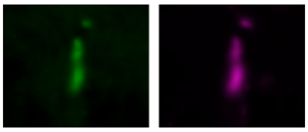

Sea otter, *Enhydra lutris lutris*
Adult, autopsy material from stranded animal. Courtesy P. Tuomi, Alaska Sea Life Center, Seward, AK, USA

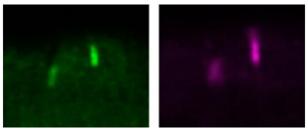

Ferret, *Mustela putorius furo*
Young animal, post mortem experimental material. Courtesy M. Leinweber, MPI for Neurobiology, Munich, Germany

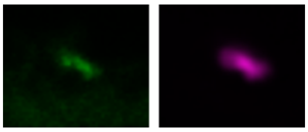

European badger, *Meles meles*
Adult, roadkill. Courtesy E. Noll, MPI for Brain Research

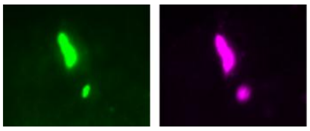

Beech marten, *Martes foina*
Adult, roadkill. Courtesy E. Noll, MPI for Brain Research

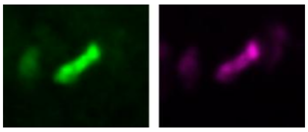

Brown bear, *Ursus arctos*
Adult hunted animal. Courtesy A. Friebe, Scandinavian Brown Bear Research Project, Kvarnberg, Sweden

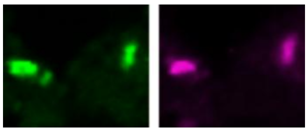

Polar bear, *Ursus (Thalarctos) maritimus*
Adult, killed for an unrelated project. Courtesy K.M. Kovacs, Norwegian Polar Institute, Tromsø, Norway

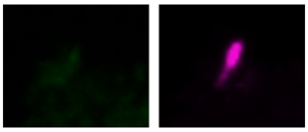

Coati, *Nasua nasua*
Adult, post mortem experimental material. Courtesy B. Pohl, Veterinary University Hannover, Germany

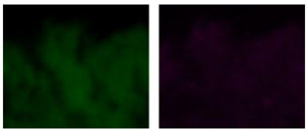

Crab-eating raccoon, *Procyon cancrivorous* (has no S1 cones[4,5])
Adult, post mortem experimental material. Courtesy B. Pohl, Veterinary University Hannover, Germany

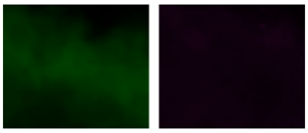

Australian Fur Seal, *Arctocephalus pusillus* (has no S1 cones[6])
Adult, autopsy material. Courtesy Taronga Zoo Sydney & U. Grünert, University of Sydney, Australia

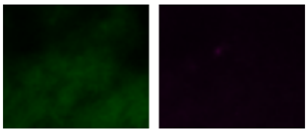

Harbour seal, *Phoca vitulina* (has no S1 cones[7,8])
2-week pup, autopsy material. Courtesy Seal Centre Friedrichskoog, Germany

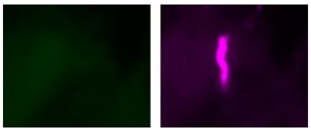

Yellow mongoose, *Cynictis penicillata*
Post mortem experimental material. Courtesy M. Manser, Inst. of Evolutionary Biology & Environmental Studies, University of Zurich, Switzerland

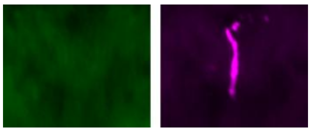

Grandidier's mongoose, *Galidictis grandidieri*
Collection material. Courtesy S.M. Goodman, Field Museum of Natural History, Chicago, IL, USA, & WWF, Madagascar

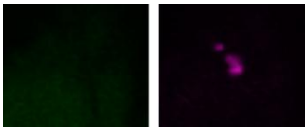

Domestic cat, *Felis catus*
Adult, post mortem experimental material, MPI for Brain Research

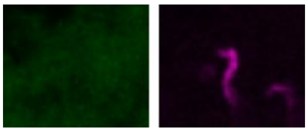

Leopard, *Panthera pardus*
Adult, autopsy material. Courtesy Veterinary Pathology Institute of Leipzig University, Leipzig, Germany

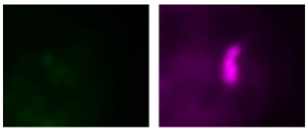

Lion, *Panthera leo leo*
Adult, autopsy material. Courtesy Veterinary Pathology Institute of Leipzig University, Leipzig, Germany

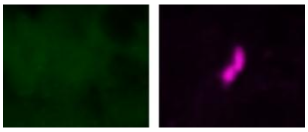

Siberian tiger, *Panthera tigris altaica*
Adult, autopsy material. Courtesy Veterinary Pathology Institute of Leipzig University, Leipzig, Germany

Perissodactyla

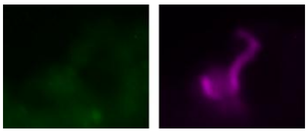

Domestic donkey, *Equus asinus asinus*
Adult, slaughterhouse material. Courtesy J. González-Soriano, Universidad Complutense, Madrid, Spain

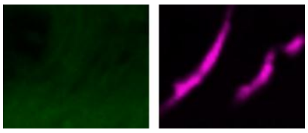

Domestic horse, *Equus caballus*
Old animal, slaughterhouse material

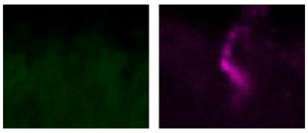

Grevy's zebra, *Equus grevyi*
Culled animal. Courtesy J. Bhattacharjee, Egerton University, Njoro, Kenya

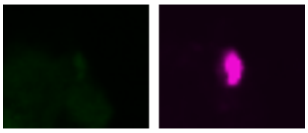

Tapir, *Tapirus terrestris*
Adult, autopsy material. Courtesy G. Wibbelt, Leibniz Institute for Zoo and Wildlife Research, Berlin, Germany

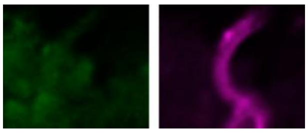

Black rhinoceros, *Diceros bicornis*
Juvenile, autopsy material. Courtesy Veterinary Pathology Institute of Leipzig University, Leipzig, Germany

Artiodactyla

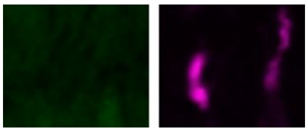

Dama gazelle, *Nanger dama*
Adult, autopsy material. Courtesy Veterinary Pathology Institute of Leipzig University, Leipzig, Germany

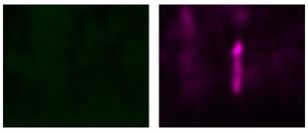

Domestic goat, *Capra aegagrus hircus*
Slaughterhouse material

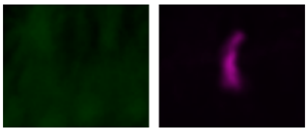

Mouflon, *Ovis orientalis*
Culled animal. Courtesy E. Noll, MPI for Brain Research

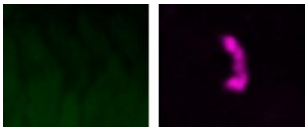

Domestic cattle, *Bos taurus*
Slaughterhouse material

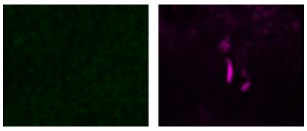

Roe deer, *Capreolus capreolus*
Adult, hunted animal. Courtesy E. Noll, MPI for Brain Research

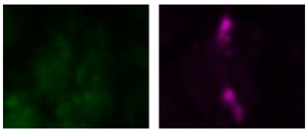

Red deer, *Cervus elaphus*
Adult, hunted animal. Courtesy E. Noll, MPI for Brain Research

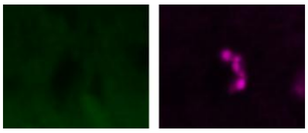

Sika deer, *Cervus nippon*
Juvenile, culled animal. Courtesy K. Burow, Wildlife Park `Alte Fasanerie', Hanau, Germany

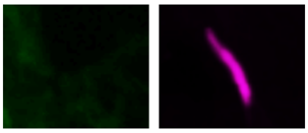

Fallow deer, *Dama dama*
Juvenile, hunted animal. Courtesy E. Noll, MPI for Brain Research

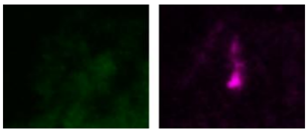

Llama, *Lama Glama*
Juvenile, autopsy material. Courtesy I. Gunsser, Ludwig Maximilians University, Munich, Germany

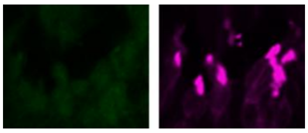

Dromedary camel, *Camelus dromedarius*
Adult, autopsy material. Courtesy G. Wibbelt, Leibniz Institute for Zoo and Wildlife Research, Berlin, Germany

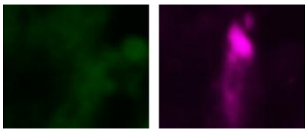

Pygmy hippopotamus, *Choeropsis liberiensis*
Adult, autopsy material. Courtesy G. Wibbelt, Leibniz Institute for Zoo and Wildlife Research, Berlin, Germany

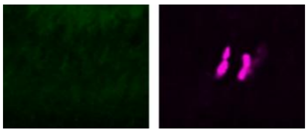

Wild boar, *Sus scrofa*
Adult, hunted animal. Courtesy E. Noll, MPI for Brain Research

Cetacea

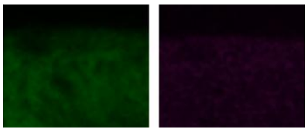

Long-finned pilot whale, *Globicephala melas* (has no S1 cones[6,9])
Courtesy G. Behrmann, Alfred Wegener Institute for Polar and Marine Research, Bremerhaven, Germany

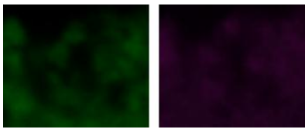

Common minke whale, *Balaenoptera acutorostrata* (has no cone opsins[9,10])
Animal killed for an unrelated project. Courtesy K.M. Kovacs, Norwegian Polar Institute, Tromsø, Norway

Table S2. Amino acid sequence of the antigen of the bird Cry1a antiserum compared with the sequences of Cry1 in different mammalian species. Identical amino acids are given in red.

Species	Cry1 labeling in retinal sections	Part of sequence	GenBank
Epitope recognized by antiserum		RPNPEEETQSVGPKVQRQST	
Monodelphis domestica (AA 565 – 584)	-	RPRQEEETQSINPKVQRQST*	XP_003342014.1
Loxodonta africana (AA 563 – 582)	-	RPSQDEETQTLGPKVQRQST*	XP_003405361.1
Macaca mulatta (AA 566 – 585)	+	RPSQEEDTQSIGPKVQRQST	NP_001181088.1
Macaca fascicularis (AA 566 – 585)	-	RPSQEEDTQSIGPKVQRQST	BAB72089.1
Mus musculus (AA 586 -604)	-	RPSQEEDAQSVGPKVQRQS	NP_031797.1
Rattus norvegicus (AA 568 – 586)	-	RPSQEEDAQSVGPKVQRQS	EDM17107.1
Cavia porcellus (AA 655-673)	-	RPSQEEDAQSTGHKIQRQS*	XP_003462370.1
Heterocephalus glaber (AA 571 – 590)	-	RPSQEEDAQSIGPKLQRQST	EHB16315.1
Oryctolagus cuniculus (AA 567 – 586)	-	RPSQEEDTQSIGPKVQRQST*	XP_002711467.1
Canis lupus familiaris (AA 567 – 586)	+	RPSEEEDTQTISPKVQRQST*	XP_862753.1
Mustela putorius furo (AA 430 – 449)	+	RPSEEEDTQSIGSKVQRQST	XP_862753.1
Felis catus domestica (AA 567 – 585)	-	RPSQEEDTQSIGPKVQRQS*	XP_003989258.1
Equus caballus (AA 567 – 586)	-	RPGPEEDTQGIGPKVQRQST*	XP_001499263.1
Bos taurus (AA 567 - 586)	-	RPSQEEDTQSIGPKVQRQST	NP_001098885.1
Sus scrofa (AA 567 – 587)	-	RPSQEEDTQSIIGPKVQRQST*	XP_003126127.1

* predicted

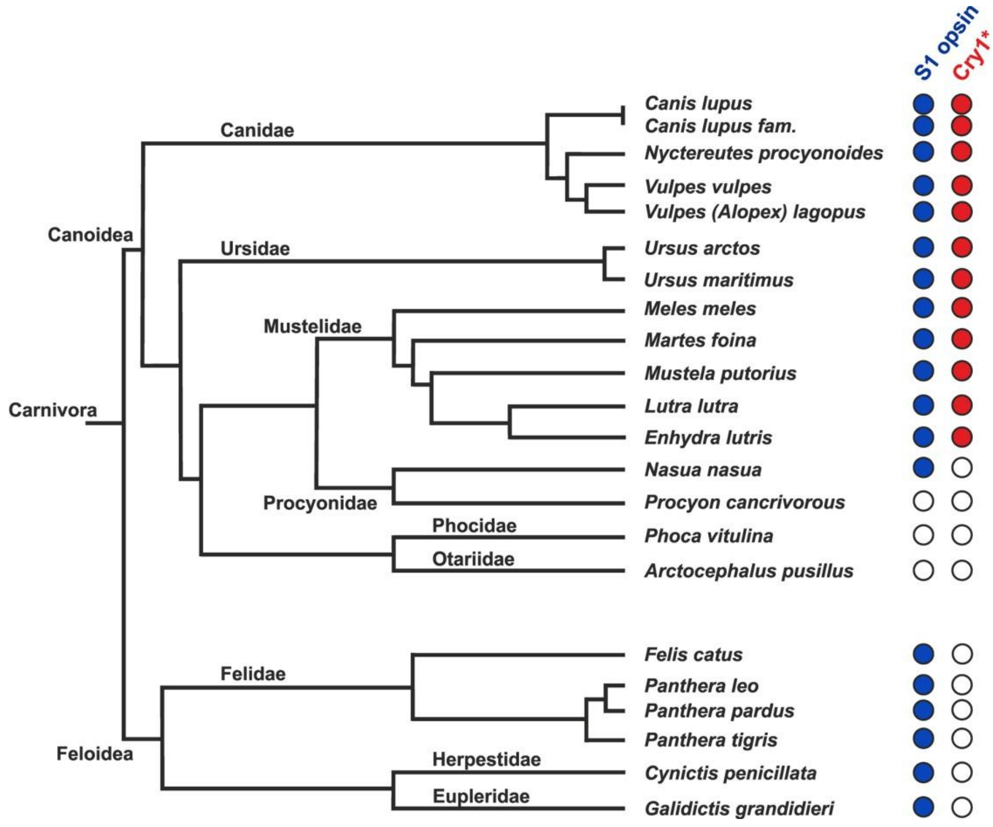

Figure S1. Phylogenetic tree for the Carnivora species studied. Simplified phylogenetic tree of the order Carnivora showing the relationships between the species tested in this study; other carnivore taxa are not included. The horizontal positions of the branch points roughly reflect the divergence times of the respective taxa. Tree data are taken from[11], where more accurate presentations of the divergence times are given. Filled coloured circles indicate the presence of S1 opsin and Cry1*, respectively, open circles indicate their absence (c.f. Supplementary Table S1).

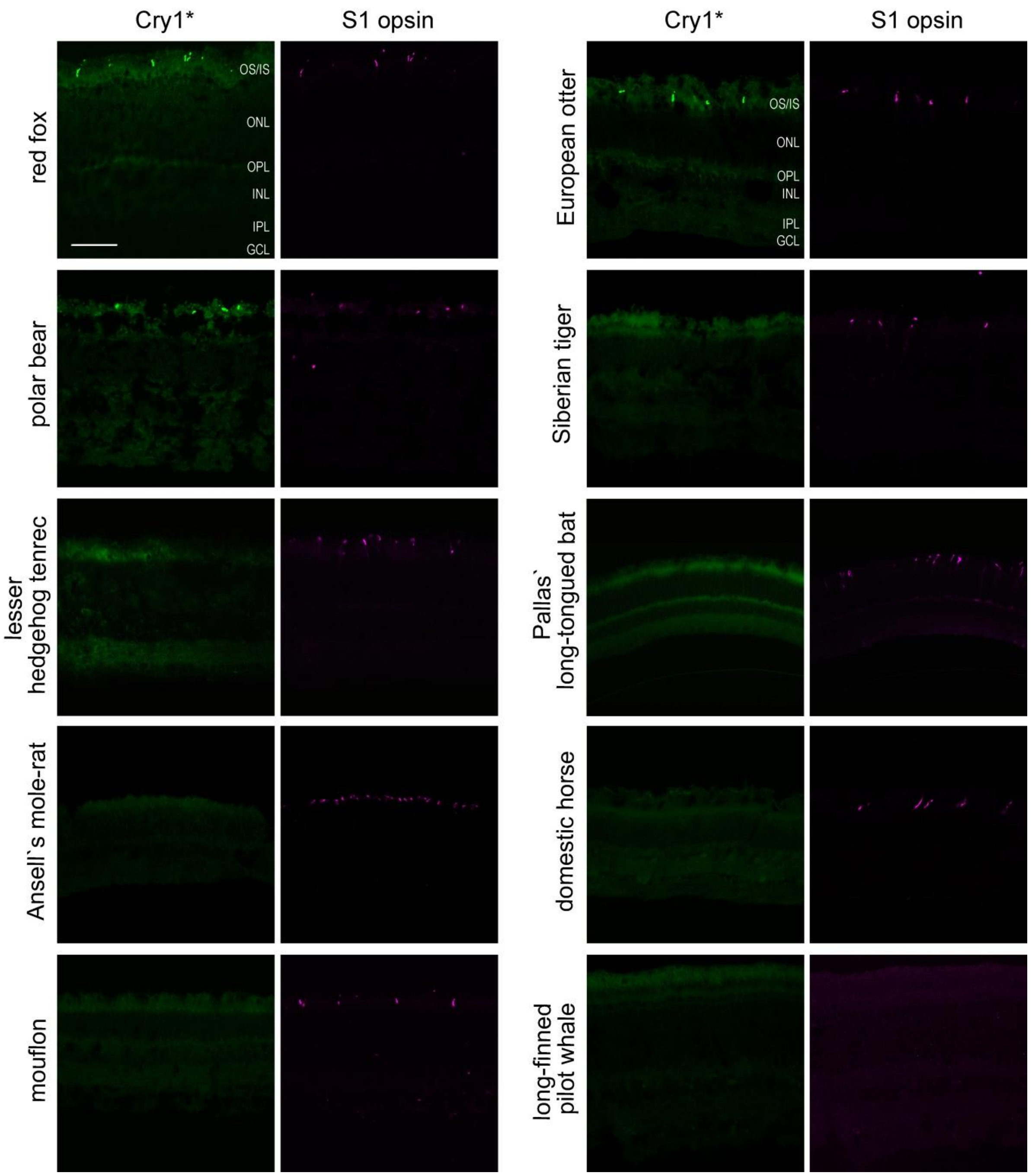

Figure S2. Examples of the retinae of different species labeled for Cry1 and S1 opsin. Vertical retinal sections of selected mammals with differing S1 opsin and Cry1* expression patterns. Each pair of images shows the same frame, exposed for Cry1* immunofluorescence (rendered in green) and S1 opsin immunofluorescence (rendered in magenta). Cry1* label is only present in the retina of red fox, European otter and polar bear, and it is restricted to the outer segments of the S1 cones. S1 opsin label is present in all illustrated species except the long-finned pilot whale, which has no S1 opsin. The retinal layers indicated exemplarily in two panels are: OS/IS, photoreceptor outer and inner segments; ONL, outer nuclear layer; OPL, outer plexiform layer; INL, inner nuclear layer; IPL, inner plexiform layer; GCL, ganglion cell layer. The scale bar represents 50 µm and applies to all panels.

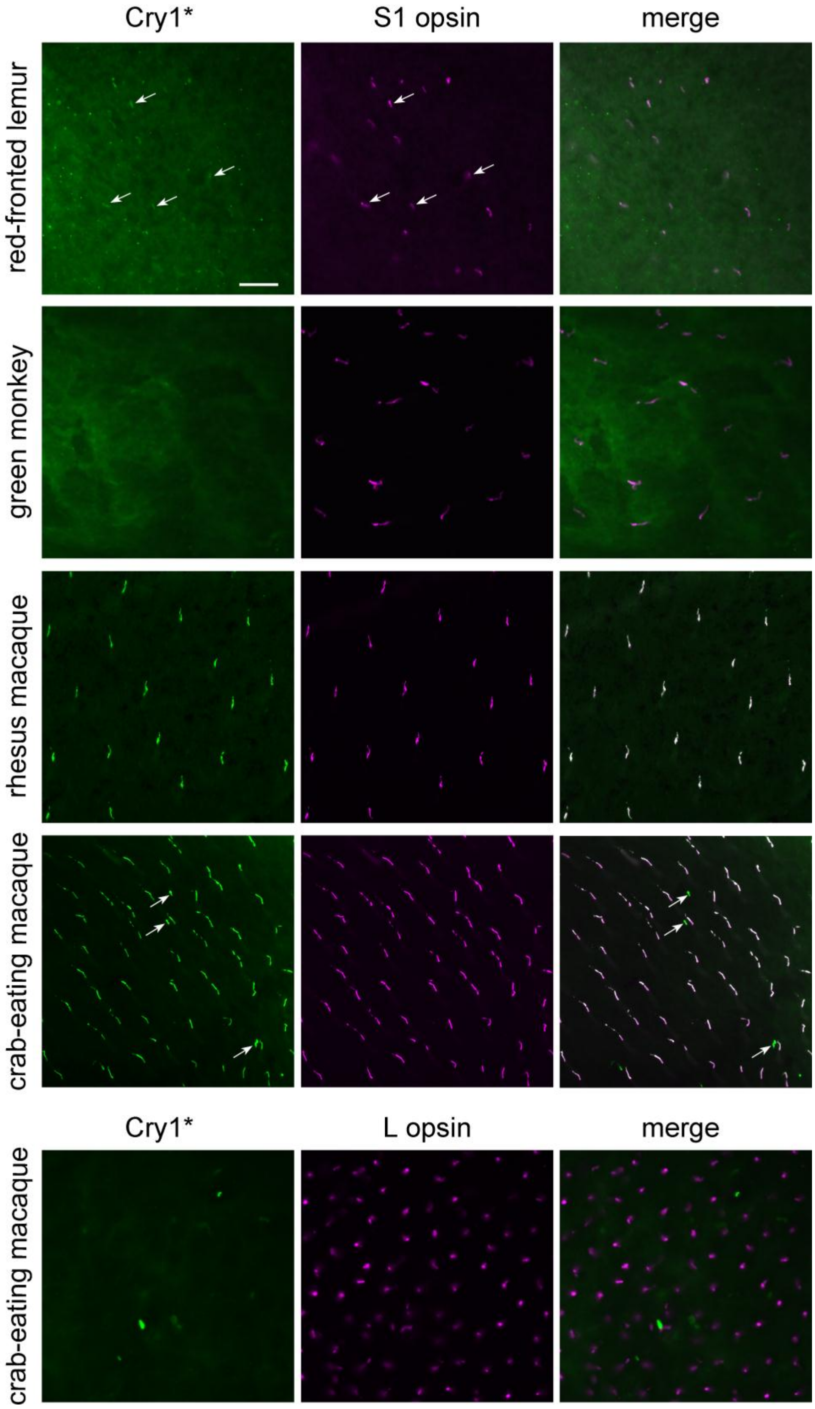

Figure S3. Examples of the retinae of primates labeled for Cry1 and S1 opsin. Retinal flatmounts double-labeled for Cry1* (left column) and S1 opsin (middle column) or L opsin (bottom row), the corresponding merges are shown in the right column. Cry1* label associated with S1 cones differs between the species. In the red-fronted lemur, Cry1* label is weak (some marked by arrows), in the green monkey no Cry1* signal is visible. In the macaques there is strong Cry1* label in the S cones, but the crab-eating macaque shows additional Cry1* label in rare structures (arrows) that are neither S1 cones nor L cones; for details see text. The scale bar represents 50 µm and applies to all panels.

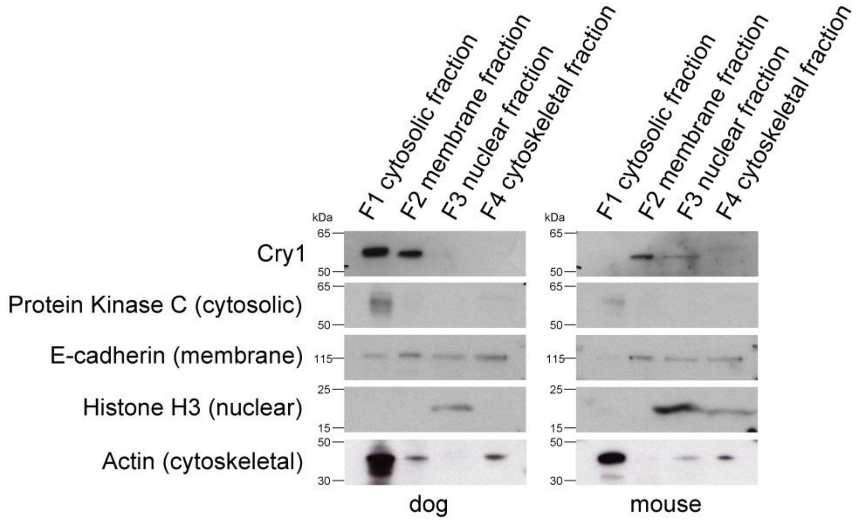

Figure S4. Western Blots of cell fractionated retinae of dog and mouse. The fractions are indicated on top, the fraction markers on the left. Cry1 is found in the cytosolic and the membrane fraction in dogs, and in the membrane and the nuclear fraction in mice. Because of increasing dissolving strength of fractionation buffers, some markers are carried over into subsequent fractions, e.g. E-cadherin or H3. Actin is present in cytosolic (probably reflecting monomeric protein) as well as cytoskeletal fractions.

Supplementary References

1. Wikler, K. C. & Rakic, P. Distribution of photoreceptor subtypes in the retina of diurnal and nocturnal primates. *J. Neurosci.* **10,** 3390-3401 (1990).
2. Ahnelt, P. K., Moutairou, K., Glösmann, M. & Kübber-Heiss, A. Lack of S-opsin expression in the brush-tailed porcupine (*Atherurus africanus*) and other mammals. Is the evolutionary persistence of S-cones a paradox? In *Normal and Defective Colour Vision* (eds Mollon J. D. *et al.*), pp. 31-38. (Oxford University Press, 2003).
3. Müller, B., Goodman, S. M. & Peichl, L. Cone photoreceptor diversity in the retinas of fruit bats (Megachiroptera). *Brain Behav. Evol.* **70,** 90-104 (2007).
4. Jacobs, G. H. & Deegan II, J. F. Cone photopigments in nocturnal and diurnal procyonids. *J. Comp. Physiol. A* **171,** 351-358 (1992).
5. Peichl, L. & Pohl, B. Cone types and cone/rod ratios in the crab-eating raccoon and coati (Procyonidae). *Invest. Ophthalmol. Vis. Sci.* **41,** 494, Abstract no. 2630 (2000).
6. Peichl, L., Behrmann, G. & Kröger, R. H. For whales and seals the ocean is not blue: a visual pigment loss in marine mammals. *Europ. J. Neurosci.* **13,** 1520-1528 (2001).
7. Peichl, L. & Moutairou, K. Absence of short-wavelength sensitive cones in the retinae of seals (Carnivora) and African giant rats (Rodentia). *Europ. J. Neurosci.* **10,** 2586-2594 (1998).
8. Crognale, M. A., Levenson, D. H., Ponganis, P.P., Deegan II, J. F. & Jacobs, G. H. Cone spectral sensitivity in the harbor seal (*Phoca vitulina*) and implications for color vision. *Can. J. Zool.* **76,** 2114-2118 (1998).
9. Meredith, R. W., Gatesy, J., Emerling, C. A., York, V. M. & Springer, M. S. Rod monochromacy and the coevolution of Cetacean retinal opsins. *PLoS Genetics* **9**(4)**,** e1003432 (2013).
10. Levenson, D. H. & Dizon, A. Genetic evidence for the ancestral loss of short-wavelength-sensitive cone pigments in mysticete and odontocete cetaceans. *Proc. R. Soc. Lond.* B **270,** 673–679 (2003).
11. Nyakatura, K. & Bininda-Emonds, O. R. P. Updating the evolutionary history of Carnivora (Mammalia): a new species-level supertree complete with divergence time estimates. *BMC Biology* **10,** 12 (2012).
